# Somatic *PIK3R1* variation as a cause of vascular malformations and overgrowth

**DOI:** 10.1038/s41436-021-01211-z

**Published:** 2021-05-26

**Authors:** Catherine E. Cottrell, Nicole R. Bender, Michael T. Zimmermann, Jonathan W. Heusel, Meagan Corliss, Michael J. Evenson, Vincent Magrini, Donald J. Corsmeier, Matthew Avenarius, Jeffrey N. Dudley, Jennifer J. Johnston, Marjorie J. Lindhurst, Katinka Vigh-Conrad, Olivia M. T. Davies, Carrie C. Coughlin, Ilona J. Frieden, Megha Tollefson, Andrea L. Zaenglein, Heather Ciliberto, Laura L. Tosi, Robert K. Semple, Leslie G. Biesecker, Beth A. Drolet

**Affiliations:** 1 The Steve and Cindy Rasmussen Institute for Genomic Medicine at Nationwide Children’s Hospital, Columbus, OH USA; 2 Department of Pathology, The Ohio State University, Columbus, OH USA; 3 Department of Dermatology, University of Florida, Gainesville, FL USA; 4 Bioinformatics Research and Development Laboratory, Genomic Sciences and Precision Medicine Center, Medical College of Wisconsin, Milwaukee, WI USA; 5 Clinical and Translational Sciences Institute, Medical College of Wisconsin, Milwaukee, WI USA; 6 Department of Biochemistry, Medical College of Wisconsin, Milwaukee, WI USA; 7 Department of Pathology & Immunology, Washington University School of Medicine, Saint Louis, MO USA; 8 Department of Genetics, Washington University School of Medicine, Saint Louis, MO USA; 9 Department of Pathology and Laboratory Medicine, The Ohio State University Wexner Medical Center, Columbus, OH USA; 10 Center for Precision Health Research, National Human Genome Research Institute, Bethesda, MD USA; 11 Division of Genetics, Oregon National Primate Research Center, Oregon Health & Science University, Portland, OR USA; 12 Medical College of Wisconsin, Milwaukee, WI USA; 13 Division of Dermatology, Departments of Medicine and Pediatrics, Washington University School of Medicine, Saint Louis, MO USA; 14 Department of Dermatology, University of California-San Francisco, San Francisco, CA USA; 15 Departments of Dermatology and Pediatrics, Mayo Clinic, Rochester, MN USA; 16 Dermatology and Pediatrics, Penn State Hershey Medical Center, Hershey, PA USA; 17 Town Square Dermatology, Coralville, IA USA; 18 Division of Orthopaedics & Sports Medicine, Children’s National Hospital, Washington, DC USA; 19 Centre for Cardiovascular Science, University of Edinburgh, Edinburgh, United Kingdom; 20 University of Wisconsin-Madison School of Medicine and Public Health, Madison, WI USA; 21 Present Address: University of Michigan Medical School, Ann Arbor, MI USA

## Abstract

**Purpose:**

Somatic activating variants in the PI3K-AKT pathway cause vascular malformations with and without overgrowth. We previously reported an individual with capillary and lymphatic malformation harboring a pathogenic somatic variant in *PIK3R1*, which encodes three PI3K complex regulatory subunits. Here, we investigate *PIK3R1* in a large cohort with vascular anomalies and identify an additional 16 individuals with somatic mosaic variants in *PIK3R1*.

**Methods:**

Affected tissue from individuals with vascular lesions and overgrowth recruited from a multisite collaborative network was studied. Next-generation sequencing targeting coding regions of cell-signaling and cancer-associated genes was performed followed by assessment of variant pathogenicity.

**Results:**

The phenotypic and variant spectrum associated with somatic variation in *PIK3R1* is reported herein. Variants occurred in the inter-SH2 or N-terminal SH2 domains of all three PIK3R1 protein products. Phenotypic features overlapped those of the PIK3CA-related overgrowth spectrum (PROS). These overlapping features included mixed vascular malformations, sandal toe gap deformity with macrodactyly, lymphatic malformations, venous ectasias, and overgrowth of soft tissue or bone.

**Conclusion:**

Somatic *PIK3R1* variants sharing attributes with cancer-associated variants cause complex vascular malformations and overgrowth. The *PIK3R1*-associated phenotypic spectrum overlaps with PROS. These data extend understanding of the diverse phenotypic spectrum attributable to genetic variation in the PI3K-AKT pathway.

## INTRODUCTION

Vascular malformations and the overgrowth syndromes of which they are commonly a part constitute a heterogeneous group of congenital malformations that lead to significant morbidity and disfigurement. Next-generation sequencing (NGS) has become an important tool in genomic investigation of these syndromes, allowing improved molecular characterization and diagnosis. Developments in NGS technology have enabled successful discovery of disease-associated somatic variation within affected tissue.^1,2^ It is now recognized that vascular malformations and overgrowth demonstrate some shared genetic variation with cancer, with the phosphoinositide 3-kinase (PI3K)-AKT growth signaling pathway commonly dysregulated in both sets of disease.^3–5^

Class I phosphatidylinositol 3-kinases (PI3K) function as heterodimers composed of a catalytic and a regulatory subunit and serve as intracellular signal transducers that convert phosphoinositide (4,5)-bisphosphate into phosphoinositide (3,4,5)-trisphosphate (PIP3). PIP3 generation triggers activation of downstream effectors including PDK1 and then AKT, which promote cell growth and survival.^6^ Somatic mosaic activating variants in *PIK3CA*, encoding the p110α catalytic subunit of the PI3K heterodimer, have been well described in vascular malformations and overgrowth syndromes.^7^ Among the regulatory subunits of PI3K, *PIK3R1* encodes three distinct protein products (p85α, p55α and p50α), generated through alternative splicing. These products form obligate heterodimers with PIK3CA, stabilizing and inhibiting it in the basal state, while mediating its binding to activated receptor tyrosine kinases and its subsequent activation.^8^ PIK3R1 also negatively regulates the PI3K pathway by stabilizing the phosphatase PTEN, itself a tumor suppressor.^9^

*PIK3R1* variants that fail to inhibit p110α activity, usually by disruption of the inter-SH2 domain, cause constitutive PI3K pathway activation, and are enriched in cancers, albeit much less commonly than *PIK3CA* variants. Given the numerous variants identified in PI3K pathway components (*PIK3CA*, *AKT1*, and *PTEN*) in overgrowth, *PIK3R1* is an excellent candidate gene for vascular malformations and overgrowth. We used targeted, high-depth NGS to analyze Affected tissue from a cohort of individuals with vascular malformations, and thereby extend understanding of the role played by *PIK3R1* in these clinically important disorders.

## MATERIALS AND METHODS

### Study cohort

The study was authorized by the institutional review board of participating institutions. Individuals described herein were identified to harbor a variant in *PIK3R1* amid the setting of apparently mosaic disease and were derived from one of three cohorts that together enabled the assemblage of variant and phenotype data within this described study cohort. The primary cohort is a multisite network coordinated through the Pediatric Dermatology Research Alliance (PeDRA)^3^ enrolling patients of any age with vascular anomalies and overgrowth. Specimens were available on 108 patients consisting of 3–4 mm skin punch biopsy samples from affected tissue or a paraffin-embedded sample of affected tissue from previous excisions. Within the PeDRA cohort, ten *PIK3R1* variant–positive individuals were identified. Among a National Institutes of Health (NIH) cohort of 297 individuals ascertained for heterogeneous manifestations of vascular anomalies and overgrowth, two *PIK3R1* variant–positive individuals were identified. The NIH cohort eligibility criteria were apparently mosaic (segmental) overgrowth of extra–central nervous system (CNS) organs/tissue. Individuals may or may not have had CNS manifestations, but those with CNS manifestations alone were not included, nor were those with vascular anomalies alone without other manifestations of overgrowth. Samples collected for sequencing included either punch skin biopsies of apparently affected tissues or excisional biopsies collected at the time of surgery. Of necessity, these samples were highly heterogeneous in nature and number, based on the clinical needs and limitations of the individuals. The Genomics and Pathology Services (GPS) at Washington University School of Medicine cohort consisted of five *PIK3R1* variant–positive individuals ascertained from a total of 343 individuals assayed clinically for suspected disorders of somatic mosaicism, including but not limited to overgrowth and vascular malformation. Where available, clinical data were retrospectively collected and included medical history, dermatologic and musculoskeletal exams, and clinical and radiologic images. Available clinical and radiologic images were reviewed centrally. The NIH study was reviewed and approved by the National Human Genome Research Institute (NHGRI) institutional review board (IRB), protocol number 94-HG-0132.

### Sequencing methodologies

NGS was utilized in all centers to identify genetic variation (Supplemental methods). DNA was extracted from affected fresh frozen tissue (FT), cultured tissue (CT), or from formalin-fixed paraffin-embedded (FFPE) blocks of previously excised affected tissue.

## RESULTS

### Identification of pathogenic variants in *PIK3R1*

**Fig. 1:**
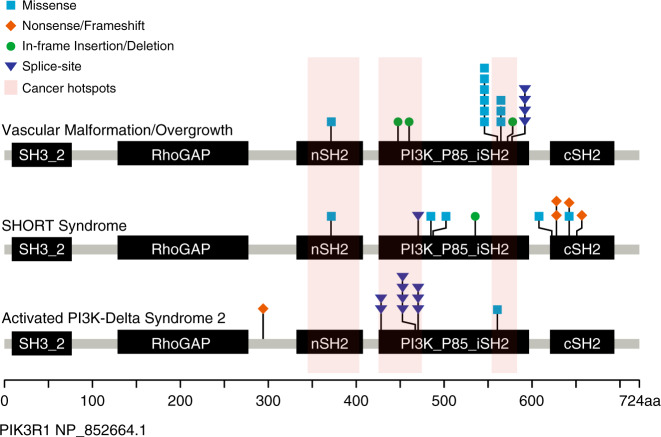
Domain structure of the longest PIK3R1 protein product showing distribution of monogenic disease-associated variants. Variants described in this study in association with a vascular malformation/overgrowth phenotype (top row). Variants described as pathogenic or likely pathogenic in the ClinVar database (accessed 13 November 2020 and filtered to encompass only variation less than 51 bp and with a described genetic condition) associated with SHORT syndrome (middle row) or activated PI3K-delta syndrome 2 (bottom row).

*PIK3R1* variants were detected in tissue of 17 individuals within the study cohort, and were observed at a reduced variant allele frequency/fraction (VAF) consistent with a somatic etiology, with most detected at less than 10% (Table 1). The identified variants included missense and insertion–deletion (indel) variants within the SH2 (*n* = 1) and PI3K_P85_iSH2 (*n* = 16) domains of PIK3R1 and overlapped regions harboring known hotspots seen in cancers (Fig. 1).^10–12^ Notably, the variants detected in vascular malformation and overgrowth occur in domains that are common to all PIK3R1 products (p85α, p55α, and p50α). Recurrent variation was detected among this cohort resulting in nine unique variants at the level of the coding sequence, and seven unique variants at the level of the predicted protein consequence. Indel events comprised three in-frame deletions and four splice-site alterations.

**Table 1:** *PIK3R1* Variant Characteristics.

*PIK3R1* coding variant (NM_181523.3)	Predicted protein consequence	Protein domain	Tissue type	Tissue source	VAF; Total NGS read depth at variant position	IND
c.1126G>A	p.(Gly376Arg)	SH2	FT	PB; reticulated port wine stain of left leg	2.9; 2,772	13
c.1355_1365delinsTTCAAGAAAAAAGTTTCTTGAAA	p.(Tyr452_Gln455 delinsPheGlnGluLysSerPheLeuLys)	PI3K_P85_iSH2	FFPE	EB; VM with features of AVM involving the epidermis/regional fibroadipose tissue on dorsum of left foot	6.4; 1,086	17
c.1392_1403delTAGATTATATGA	p.(Asp464_Tyr467del)	PI3K_P85_iSH2	FT	Affected skin	1.5; 1,290	14
c.1690A>G	p.(Asn564Asp)	PI3K_P85_iSH2	CT	Affected tissue	39.8^a^; 1,224	1
c.1690A>G	p.(Asn564Asp)	PI3K_P85_iSH2	FT	Affected tissue	24; 321	2
c.1690A>G	p.(Asn564Asp)	PI3K_P85_iSH2	FFPE	EB; soft tissue mass consistent with a benign vascular malformation of the left forearm	3.4; 1,980	3
c.1690A>G	p.(Asn564Asp)	PI3K_P85_iSH2	FT	PB; affected skin	2.8; 2,779	10
c.1690A>G	p.(Asn564Asp)	PI3K_P85_iSH2	FT	PB; affected skin	2.2; 4,159	12
c.1690A>G	p.(Asn564Asp)	PI3K_P85_iSH2	FT	PB; affected skin	1.8; 6,616	16
c.1699A>G	p.(Lys567Glu)	PI3K_P85_iSH2	FT	PB; affected skin	4.1; 2,675	4
c.1699A>G	p.(Lys567Glu)	PI3K_P85_iSH2	FT	PB; affected skin	2.3; 2,737	9
c.1699A>G	p.(Lys567Glu)	PI3K_P85_iSH2	FT	PB; affected skin	1.1; 1,425	11
c.1735_1740delCAATAC	p.(Gln579_Tyr580del)	PI3K_P85_iSH2	FT	PB; affected skin	13.3; 1,832	5
c.1746–6_1751delTTTCAGGTGGTT	p.(Met582_Asp605delinsIle)	PI3K_P85_iSH2	FT	PB; affected skin	3.1; 4,205	6
c.1746–5_1748delTTCAGGTG	p.(Met582_Asp605delinsIle)	PI3K_P85_iSH2	FFPE	EB; affected skin	5.9; 1,174	7
c.1748_1750delGGT	p.(Met582_Asp605delinsIle)	PI3K_P85_iSH2	FT	PB; affected skin	1.4; 2,886	8
c.1748_1750delGGT	p.(Met582_Asp605delinsIle)	PI3K_P85_iSH2	FT	PB; port wine stain of right forearm	2.2; 3,317	15

*AVM* arteriovenous malformation, *CT* cultured tissue, *EB* excisional biopsy, *FFPE* formalin-fixed paraffin-embedded tissue, *FT* fresh frozen tissue, *IND* individual, *PB* punch biopsy, *VAF* variant allele frequency/fraction, *VM* vascular malformation.

^a^For individual 1, five samples of affected tissue were assayed by next-generation sequencing (NGS) or restriction fragment length polymorphism (RFLP) studies. Tissues including skin (NGS, 39.8% VAF; RFLP, 55.6% VAF), bone (RFLP, 26.49% VAF), cartilage (RFLP, 28.1% VAF), fat (RFLP, 0.96% VAF) and a mixed sample (RFLP, 1.25% VAF) were all cultured and DNA isolated from cultured cells was analyzed. Uncultured biopsy samples were not genotyped. Blood and unaffected fibroblasts were not observed to harbor the variant.

### Variant attributes

Variants were interpreted using a modification of the American College of Medical Genetics and Genomics and the Association for Molecular Pathology (ACMG/AMP) standards and guidelines for variant interpretation as described in the Supplemental methods.^13^ Variants were considered to be of somatic origin if the variant allele was observed at a diminished VAF or a variant was present at differing frequencies among tissues from the same individual. Variant attributes considered when assessing for pathogenicity included variant location within the gene or protein product (hotspot or domain), occurrence and frequency within this cohort, and within the wider setting of human disease, as well as variant type and predicted impact on the protein product. Among the detected variants, 15 were classified as pathogenic (5 of these being unique at the level of the predicted amino acid change), with 2 classified as likely pathogenic.

### Clinical phenotype in individuals with *PIK3R1* variants

**Fig. 2:**
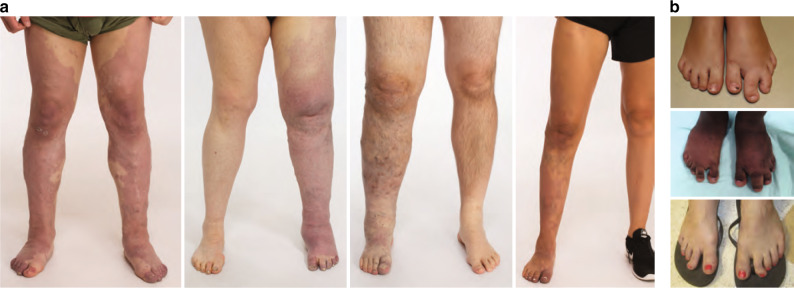
Patient photographs of characteristic clinical phenotype. (**a**) Capillary venous malformation with limb overgrowth (Klippel–Trenaunay). (**b**) Macrodactyly with sandal toe deformity.

Expert-reviewed clinical images and data were available for 12 individuals harboring *PIK3R1* variants, and their phenotypes were similar to those attributed to somatic mosaic hotspot variants in *PIK3CA*. Most patients had red vascular stains (10/12), venous ectasias or engorgement (11/12), and mild soft tissue or bone overgrowth (11/12). Four individuals had sandal toe gap deformities of the foot with mild macrodactyly of the second toe (Table 2, Fig. 2). Other clinical features noted included developmental delay, cutaneous syndactyly, and lipoma/fatty tissue overgrowth. Among individuals in whom extensive phenotype information was available (PeDRA cohort, *n* = 10; NIH cohort, *n* = 2), seizures, macrocephaly, or hydrocephalus were not described. Individuals had been previously diagnosed with various acronyms or eponyms including Klippel–Trenaunay syndrome, CLOVES syndrome, and PROS.

**Table 2:** Phenotypic Characteristics among *PIK3R1* Variant–Positive Individuals.

		Phenotypic features								
IND	Age (years)^a^	DD	CM	LM	VM	OVG	Skeletal abnormalities	Other data	*PIK3R1* coding variant (NM_181523.3)	Variant classification and type
1	10	N	Y	Y	Y	Y	Macrodactyly, sandal gap	Fatty OVG	c.1690A>G	Pathogenic; missense
2	5	N	Y	Y	Y	Y	Macrodactyly, Leg length discrepancy, sandal gap	Right congenital buphthalmos (congenital toxoplasmosis), right anterior segment dysgenesis with glaucoma, right microphthalmia	c.1690A>G	Pathogenic; missense
3	6	NP	NP	NP	NP	NP	NP	Indication: congenital malformation syndrome involving early overgrowth; concern for CLOVES	c.1690A>G	Pathogenic; missense
4^b^	17	N	Y	Y	Y	Y	Leg length discrepancy		c.1699A>G	Pathogenic; missense
5	50	N	Y	Y	Y	Y	Macrodactyly, syndactyly	Lipoma	c.1735_1740delCAATAC	Pathogenic; in-frame deletion
6	12	Y	Y	N	N	Y	N		c.1746–6_1751delTTTCAGGTGGTT	Pathogenic; splice-site
7	30	N	Y	N	Y	Y	Macrodactyly, sandal gap		c.1746–5_1748delTTCAGGTG	Pathogenic; splice-site
8	59	N	N	N	Y	Y	N		c.1748_1750delGGT	Pathogenic; splice-site
9	21	N	Y	N	Y	Y	N		c.1699A>G	Pathogenic; missense
10	18	N	Y	Y	Y	Y	N	Lipoma	c.1690A>G	Pathogenic; missense
11	51	N	Y	N	Y	Y	Leg length discrepancy		c.1699A>G	Pathogenic; missense
12	10	N	Y	Y	Y	Y	N	Frontal bossing	c.1690A>G	Pathogenic; missense
13	1	NP	NP	NP	NP	NP	NP	Indication: reticulated port wine stain of left leg also affecting left side of scrotum; left leg hypoplastic; left testicular nubbin	c.1126G>A	Pathogenic; missense
14	2	NP	NP	NP	NP	NP	NP	Indication: Klippel–Trenaunay syndrome	c.1392_1403delTAGATTATATGA	Likely pathogenic; in-frame deletion
15	20	NP	NP	NP	NP	NP	NP	Indication: vascular nevus, hemihypertrophy	c.1748_1750delGGT	Pathogenic; splice-site
16	39	N	Y	Y	Y	Y	N		c.1690A>G	Pathogenic; missense
17	15	NP	NP	NP	NP	NP	NP	Indication: Klippel–Trenaunay syndrome; vascular malformation and lipoma of left foot	c.1355_1365delinsTTCAAGAAAAAAGTTTCTTGAAA	Likely pathogenic; in-frame deletion

*DD* developmental delay, *CM * capillary malformation, *IND* individual, *LM * lymphatic malformation, *NP* not phenotyped by expert review (therefore indication for study listed in Other Data), *OVG* overgrowth, *VM* venous malformation.

^a^Individual’s age at time of enrollment in years.

^b^Individual described previously in Siegel et al.^3^

## DISCUSSION

*PIK3R1* is ubiquitously expressed and has important roles in physiology and disease. It encodes the p85α, p55α, and p50α regulatory subunits of class 1A PI3K, which bind tightly to any of the p110α, β, or δ catalytic subunits. It is the p110α subunit, encoded by *PIK3CA*, that is by far the most commonly mutated and activated in cancer and overgrowth syndromes. PI3K transduces cell surface activation of receptor tyrosine kinase growth factor and hormone receptors into downstream activation of AKT and other pathways to regulate cell metabolism, size, differentiation, proliferation, migration, and apoptosis.^14,15^ The PI3K/AKT pathway is constitutively activated in affected tissue from many vascular malformations,^1,16,17^ primarily through mosaic variation in *PIK3CA*, as in cancer.

*PIK3R1* variation in the setting of vascular malformation with overgrowth has been rarely reported. We previously described a single individual harboring a somatic mosaic *PIK3R1* variant, p.(Lys567Glu), with capillary and lymphatic malformation, and leg length discrepancy (individual 4 as referenced in this cohort).^3^ A further patient harboring a *PIK3R1* iSH2 domain variant, p.(Asn564Lys), was reported to have macrocephaly, tracheomalacia, and cardiovascular malformation described by the authors as in the context of megalencephaly–capillary malformation (MCAP) syndrome, as well as recurrent infections in keeping with activated PI3K-delta syndrome 2 (APDS2).^18^ We confirm in a large cohort that somatic mosaic *PIK3R1* variants are a significant cause of vascular malformation and overgrowth. The phenotypes observed in individuals with somatic mosaic variants in *PIK3R1* are similar to those associated with mosaic *PIK3CA* hotspot variants. All individuals with mosaic *PIK3R1* variants had red-purple, geographic vascular stains, most with associated venous engorgement, venous prominence, and soft tissue or bone overgrowth, but this tended to be mild and relatively uniform. One individual with a unique variant within the N-terminal SH2 domain, p.(Gly376Arg), had a light pink reticulate vascular stain with associated limb undergrowth. Residue 376 has been designated as a cancer hotspot, with the variant itself, p.(Gly376Arg), functionally characterized as capable of inducing in vitro oncogenic transformation and activation of p110α.^19,20^ In total, the observed clinical features among the individuals assembled within our cohort suggest that somatic mosaic variants in *PIK3R1* activate the PI3K pathway; however, the degree of activation, particularly in comparison to disease-associated variation in *PIK3CA*, requires further study.

As is the case for somatic mosaic overgrowth-associated variants in *PIK3CA* and *AKT1*, mosaic *PIK3R1* variants are also found in cancer.^21^ *PIK3CA* is more commonly altered in cancer in comparison to *PIK3R1*. Among 181 studies with nonredundant samples curated in cBioPortal encompassing in total 47,580 samples, 10.3% harbored a *PIK3CA* variant, as opposed to 2.1% for *PIK3R1* (date accessed 11 November 2020). Among this curated data set, in-frame variants were more common in *PIK3R1* (22.2% of all variants) than in *PIK3CA* (2.5% of all variants). Similarly, in our PeDRA multisite network with vascular anomalies and overgrowth, *PIK3R1* variants were less common (9.2%, 10/108) than variants in *PIK3CA* (39.8%, 43/108 individuals), with notable enrichment of in-frame variants in *PIK3R1* (Supplemental Fig. 1).

Constitutional variants in *PIK3R1* have been shown to exhibit striking genotype–phenotype correlation. Most pertinent to this study, variants disrupting canonical splicing of exon 11 and leading to in-frame deletions in the N-terminal of the inter-SH2 domain cause APDS2, an immunodeficiency characterized by recurrent infections and lymphoproliferation.^22^ Hyperactivation of PI3K signaling in APDS2 has been demonstrated in lymphocytes, yet despite ubiquitous expression of the pathogenic variant, associated overgrowth has been exceedingly rarely described.^18,23^ In vitro studies have shown that APDS2 variants in *PIK3R1* cause distinct patterns of hyperactivation of p110δ, the dominant lymphocyte catalytic subunit, and p110α, the ubiquitous growth-promoting subunit, based on subtle differences in the inhibitory molecular interactions of the regulatory and catalytic subunits: although both are hyperactivated, basal hyperactivation of p110δ was greater than 300-fold, while basal activation of p110α was only twofold.^24^ This establishes that subtle differences in molecular interactions at the dynamic interface of regulatory and catalytic subunits can have major effects on the pattern of biochemical activation of mutant holoenzyme. This has yet to be studied for vascular and overgrowth-related *PIK3R1* variants, but it is plausible that these, too, result in a distinct profile of biochemical activation of different catalytic subunits. A full account of biochemical differences will also have to address any effects of variations in PI3K subunit expression and stoichiometry in various tissues, which is known to modulate PI3K activity.

In the aforementioned individual described with features of APDS2 and MCAP, the pathogenic heterozygous *PIK3R1* variant, p.(Asn564Lys), was associated with mildly increased lymphocyte AKT phosphorylation.^18^ A different missense change at codon 564, p.(Asn564Asp), was the most frequently detected variant in our vascular malformation and overgrowth cohort and is also described in cancer. Interestingly, in biochemical studies it has been shown to increase basal activity of p110α and β significantly more than δ, a pattern opposite to that described for the APDS2 variant, albeit in a different experimental paradigm.^25^ Overt clinical manifestations of immune dysregulation were not identified in any of the individuals within our cohort, however; most patients did not undergo systematic laboratory evaluation for abnormalities in B cells and T cells.

In contrast to APDS2, constitutional *PIK3R1* loss-of-function variants, predominantly in the C-terminal SH2 domain, have been shown to cause SHORT syndrome (short stature–hyperextensibility–hernia–ocular depression–Rieger anomaly–teething delay).^26^ These variants disrupt association of PI3K holoenzyme with activated RTKs, leading to downstream hypostimulation of the PI3K/AKT axis in response to ligand stimulation. The phenotype is correspondingly the “inverse” of vascular malformations and overgrowth, including intrauterine growth restriction, lipoatrophy, and insulin resistance/diabetes.^27^ Of note, very rare reports of phenotypic overlap have been described between APDS2 and SHORT syndrome.^28^ In principle, these findings suggest that there are largely distinct spectra of *PIK3R1* variants associated with these three disorders. Biochemical studies demonstrate that differences between SHORT syndrome and APDS2 are attributable to different profiles of activation or repression of PIK3CA and PIK3CD by mutant *PIK3R1* products.

The mutational spectrum of *PIK3R1*-related somatic mosaic overgrowth is largely distinct from the constitutional *PIK3R1*-related disorders, and no apparent loss-of-function variants were seen among 17 unrelated affected individuals. Recurrent missense variants were identified, with one variant observed in affected tissue from three individuals, p.(Lys567Glu), and one variant in six individuals, p.(Asn564Asp). Indel variants were a frequent observation within this cohort with 7/17 (41%) variants of this type detected. These included three predicted in-frame events and four splice-site alterations, the latter recurrently located at the intron 13/exon 14 junction (NM_181523.3) (Supplemental Fig. 2, Supplemental Table I). Due to the genomic architecture in this region, codon 582, which encodes a methionine, is split across exons 13 and 14. Furthermore, the intron 13/exon 14 junction consists of a spanning GGT sequence that is repeated once in exon 14. Genomic complexity and variability in published variant descriptions within this region further confound interpretation. Based solely on the observed sequencing data and established bioinformatic and nomenclature conventions, we would describe one such variant, c.1748_1750del, as predicted to encode p.(Trp583del). Notably, this variant has been reported previously in cancer literature and databases described as Trp583del, or as a splice variant affecting methionine codon 582.^10,29,30^ In cancer studies, RNA sequencing demonstrates that variably sized indel splice variants impacting the splice acceptor (described as M582_splice) result in in-frame exon 14 skipping.^11^ Based on these data, the protein consequence of these exon 14 skipping variants should be described as p.(Met582_Asp605delinsIle).

Both simple and complex indels are enriched in *PIK3R1* at recurrent genomic regions defined as hotspots occurring within discrete clusters within the SH2 and inter-SH2 domains, with demonstrated statistical significance observed from large cancer data sets (Supplemental Table I) (cancerhotspots.org).^11,12,31^ One such variant in our cohort, p.(Gln579_Tyr580del), has been shown to demonstrate nearly twofold increased activity as measured by an in vitro phosphatidylinositol phosphorylation assay, retaining p110α binding but losing inhibitory activity.^25,32^ Further studies to establish the activation pattern conferred by *PIK3R1* variants associated with vascular malformations and overgrowth are needed. Moreover, characterization of *PIK3R1* variants and functional impact are of particular importance in consideration of treatment. The application of targeted therapeutics has been previously investigated in the setting of vascular malformation and somatic overgrowth. Importantly, studies of alpelisib, a targeted inhibitor of p110α, have demonstrated efficacy in PROS with attenuation of disease symptomatology and may reasonably be considered for further study in the setting of *PIK3R1* variation.^33^

The somatic mosaic *PIK3R1* variants we describe in vascular malformations and overgrowth further extend our understanding of *PIK3R1*, whose genetic perturbation produces pleiotropic manifestations. Several features of the vascular phenotype are the reverse of SHORT syndrome, displaying vascular and soft tissue overgrowth as opposed to short stature, reduced adipose tissue, and tissue underdevelopment observed in SHORT syndrome. Divergent clinical phenotypes are determined by the nature of the *PIK3R1* alteration, the specificity of the consequences for PIK3Cα- and PIK3Cδ-mediated effects, and the timing and distribution of the alteration during embryogenesis. Disease-associated *PIK3R1* variants are enriched for indel events in both cancer and vascular anomalies with overgrowth. Detection of such variants is bioinformatically challenging, and further complicated by nonstandardized annotation of the variant nomenclature. As such, these indel events are subject to ascertainment bias with additional study needed to discern frequency. Furthermore, studies to elucidate the molecular mechanisms underlying pathogenic variation of *PIK3R1* with widely disparate clinical phenotypes, including both under- and overgrowth syndromes and immunoregulation, will unify our understanding of this critical cellular proliferation pathway and provide further insight into treatment.

## Supplementary information

Supplementary Information

Supplementary Table

## Data Availability

Variant data have been deposited into the ClinVar database (https://www.ncbi.nlm.nih.gov/clinvar/) with the following submission identifiers: SCV001478395, SCV001478396, SCV001478397, SCV001478398, SCV001478399, SCV001478400, SCV001478401, SCV001478402, SCV001478403.
