# Supplementary material for: Somatic PIK3R1 variation as a cause of vascular malformations and overgrowth
Source: Genet Med. 2021 May 26;23(10):1882–8. doi: 10.1038/s41436-021-01211-z (PMC8486672; doi:10.1038/s41436-021-01211-z)
Supplement: Supplementary file 1 — Supplementary Information [file 41436_2021_1211_MOESM1_ESM.pdf]

Supplementary Material

Methodology

Primary and Washington University School of Medicine Cohorts

Sequencing studies of the primary cohort (consisting of a 16-institution collaborative network), and the Washington University School of Medicine cohort encompassed the methodologies described herein. Sequencing libraries were created following acoustic-focused fragmentation (Covaris), DNA end-repair, A-tailing, and indexing using the KAPA HyperPrep Kit (Roche Sequencing and Life Science KAPA Biosystems, Wilmington, MA) or Agilent SureSelect Library Kit (Agilent, Santa Clara, CA). Adapter-ligated DNA was subjected to limited-cycle amplification prior to target enrichment with either custom DNA or RNA capture probes (Integrated DNA Technologies, Coralville, IA or Agilent, respectively). Target capture space encompassed between 390 kb and 682 kb depending on assay version, ranging between 131-177 genes enriched for loci involved in cell signaling, oncogenesis and tumor suppression.

High-depth massively parallel sequencing was performed on Illumina instrumentation (Ilumina, Inc., San Diego, CA) to obtain short paired-end reads (101 or 151 bp) with average, unique on-target read depths of >1,000X across the capture space for high sensitivity in variant calling.

To orthogonally verify somatic variants at low allele frequency observed in *PIK3R1* in the primary cohort, two samples with available residual nucleic acid (representing disease-involved tissue from individual 4 and individual 6) were utilized for high-depth amplicon sequencing. *PIK3R1* primers were designed to encompass both the previously detected single nucleotide variant (SNV) in individual 4 and small deletion in individual 6 within a 296 bp PCR product: Forward primer 5'AGAAGCAGGCAGCTGAGTAT (GRCh37 chr5:67,591,056-67,591,075) and reverse primer 5'ATCTTCTGCTATCACCATCTTT (GRCh37 chr5:67,591,330-67,591,351). These amplified products were size confirmed, ligated with dual-match Illumina adapters (AGAAGGAC and AGAAGCCT, respectively) and re-amplified with Illumina universal P5/P7 primers. The amplicons were diluted to 50fM and sequenced on the Illumina MiniSeq.

Following massively parallel sequencing of captured libraries, BAM files were visualized using the Integrative Genomics Viewer[1] and demonstrated the expected SNV (c.1699A>G) and deletion event (c.1746-6_1751delTTTCAGGTGGTT) harbored by individuals 4 and 6, respectively. The c.1699A>G substitution (individual 4) was supported by 85 reads out of 2,259 total reads (3.8%). Amplicon sequencing data on individual 6 demonstrated reduced read support of 1526 averaged reads observed across positions chr5:67591239-67591250 representing the left-aligned deletion event in the BAM file. Immediately flanking this depressed read count value at chr5:67591238 and chr5:67591251, coverage increased to 1,655 reads supporting the small 12 bp deletion present at frequency of 7.8%.

The National Institutes of Health Cohort

The individuals evaluated at the NIH were first screened with a restriction fragment length polymorphism (RFLP) panel that tests for four common variants in *PIK3CA* and the c.49G>A p.(E17K) *AKT1* variant. RFLP negative samples were then reflexed to a custom capture next-generation sequencing (NGS) panel that interrogated >250 genes, including *PIK3R1,* and sequenced on an Illumina MiSeq instrument. The c.1690A>G variant in *PIK3R1* detected in individuals 1 and 2 was confirmed using a custom RFLP assay.

Variant Interpretation

Applicable criteria for the scoring of mosaic variation described within this cohort was adapted from Richards et al. as determined by CEC and LGB.[2]

<u>Strong Evidence of Pathogenicity Criteria:</u>

PS2

The observation of apparent somatic mosaicism was considered in the interpretation schema as applied to the existing de novo criteria in the American College of Medical Genetics and Genomics and the Association for Molecular Pathology (ACMG/AMP) standards and guidelines for variant interpretation.[2] Variants were considered to be of somatic etiology if a variant allele frequency/fraction (VAF) ranging between 1-25% was detected in a given sample or if a variant was present at differing frequencies among studied tissues from the same individual. Variants detected with a VAF of ≥ 3% as determined at the level of the coding variant (among any tested sample within the cohort in the case of recurrent coding variation), with no discernable strand bias, in regions absent of repetition, and sequence homology[3] and with clean, high-quality reads were considered as de novo events meeting strong criteria (PS2). Those variants at <3% VAF were considered as a moderate level of support of *de novo* status (PS2_Mod).

PS_Cancer

A novel strong criterion (PS_CANCER) was applied if the variant was well-represented in cancer as identified in the COSMIC database with ≥20 documented instances (cancer.sanger.ac.uk)[4] and considered to occur in a statistically significant hotspot or region (cancerhotspots.org)[5,6] within *PIK3R1*. This criterion was considered a moderate level of evidence if only one of the qualifiers (COSMIC or cancer hotspot) was met.

PS3

Functional studies were applied on the basis of available data in the literature using well-established models demonstrating downstream impact of the variant on RNA structure, gene expression or protein function.

<u>Moderate Evidence of Pathogenicity Criteria:</u>

PS4_Mod

At the level of the predicted amino acid change, the prevalence of the variant within our combined cohort was considered as a moderate level of evidence as applied in the setting of PS4 (prevalence of the variant in affected individuals is significantly increased compared with the prevalence in controls in the absence of a defined relative risk or odds ratio), with the observation of ≥5 unique occurrences in our cohort. PS4 was further downgraded to a supporting level of evidence where <5 and ≥3 unrelated individuals harbored the same predicted amino acid change within our cohort.

PM4

A moderate level of evidence criterion (PM4) was applied for protein length changes related to insertion/deletion events in a non-repeat region predicted to result in an in-frame protein product.

Supporting Evidence of Pathogenicity Criteria:

PP2

The criterion PP2 was applied for all *PIK3R1* missense variants due to constraint against missense changes in the gene, in the setting of a low rate of benign missense variation, given that missense changes are a common mechanism of both cancer and constitutional disease.

Criteria Not Applied:

PM2

Given the nature of the somatic variation under study, PM2 (absent from control or population databases) was not formally applied as evidence used in the setting of variant classification.

PP3

In silico prediction scores (PP3) were not applied due to the discordance in algorithms adequately identifying activating alterations.

References

1. Robinson JT, Thorvaldsdóttir H, Winckler W, et al. Integrative genomics viewer. *Nat Biotechnol.* Jan 2011;29(1):24-26.
2. Richards S, Aziz N, Bale S, et al. Standards and guidelines for the interpretation of sequence variants: a joint consensus recommendation of the American College of Medical Genetics and Genomics and the Association for Molecular Pathology. *Genetics in Medicine.* 2015/05/01 2015;17(5):405-423.
3. Mandelker D, Schmidt RJ, Ankala A, et al. Navigating highly homologous genes in a molecular diagnostic setting: a resource for clinical next-generation sequencing. *Genet Med.* Dec 2016;18(12):1282-1289.
4. Tate JG, Bamford S, Jubb HC, et al. COSMIC: the Catalogue Of Somatic Mutations In Cancer. *Nucleic acids research.* Jan 8 2019;47(D1):D941-d947.
5. Chang MT, Asthana S, Gao SP, et al. Identifying recurrent mutations in cancer reveals widespread lineage diversity and mutational specificity. *Nat Biotechnol.* Feb 2016;34(2):155-163.
6. Gao J, Chang MT, Johnsen HC, et al. 3D clusters of somatic mutations in cancer reveal numerous rare mutations as functional targets. *Genome Med.* Jan 23 2017;9(1):4.
7. Cerami E, Gao J, Dogrusoz U, et al. The cBio cancer genomics portal: an open platform for exploring multidimensional cancer genomics data. *Cancer discovery.* May 2012;2(5):401-404.
8. Gao J, Aksoy BA, Dogrusoz U, et al. Integrative analysis of complex cancer genomics and clinical profiles using the cBioPortal. *Science signaling.* Apr 2 2013;6(269):pl1.

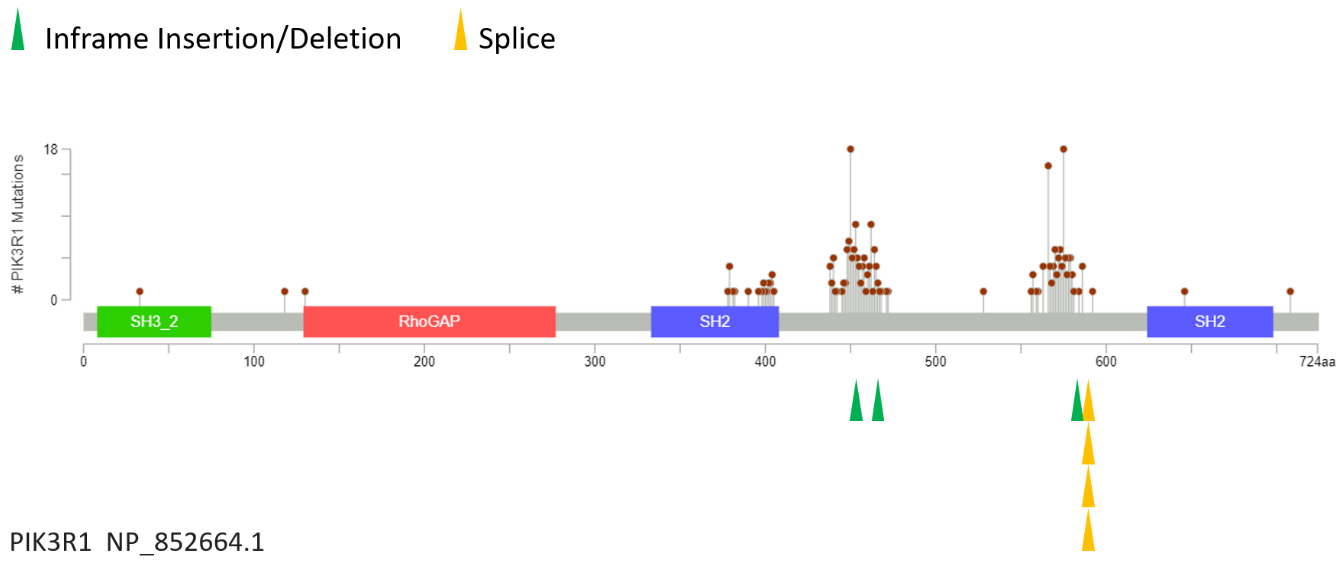

Supplementary Figure 1: Schematic Representation of *PIK3R1* Variants in Cancer and Vascular Malformation/Overgrowth

Plot demonstrating the frequency and location of insertion/deletion (indel) events in *PIK3R1* among a curated set of 181 non-redundant studies consisting of 47,580 cancer samples visualizable within cBioPortal (accessed 11-11-2020).[7,8] Indel hotspot events are clustered within the SH2 and inter-SH2 domains. Triangles highlight the frequency and location of indels among our multi-site cohort with vascular anomalies and overgrowth.

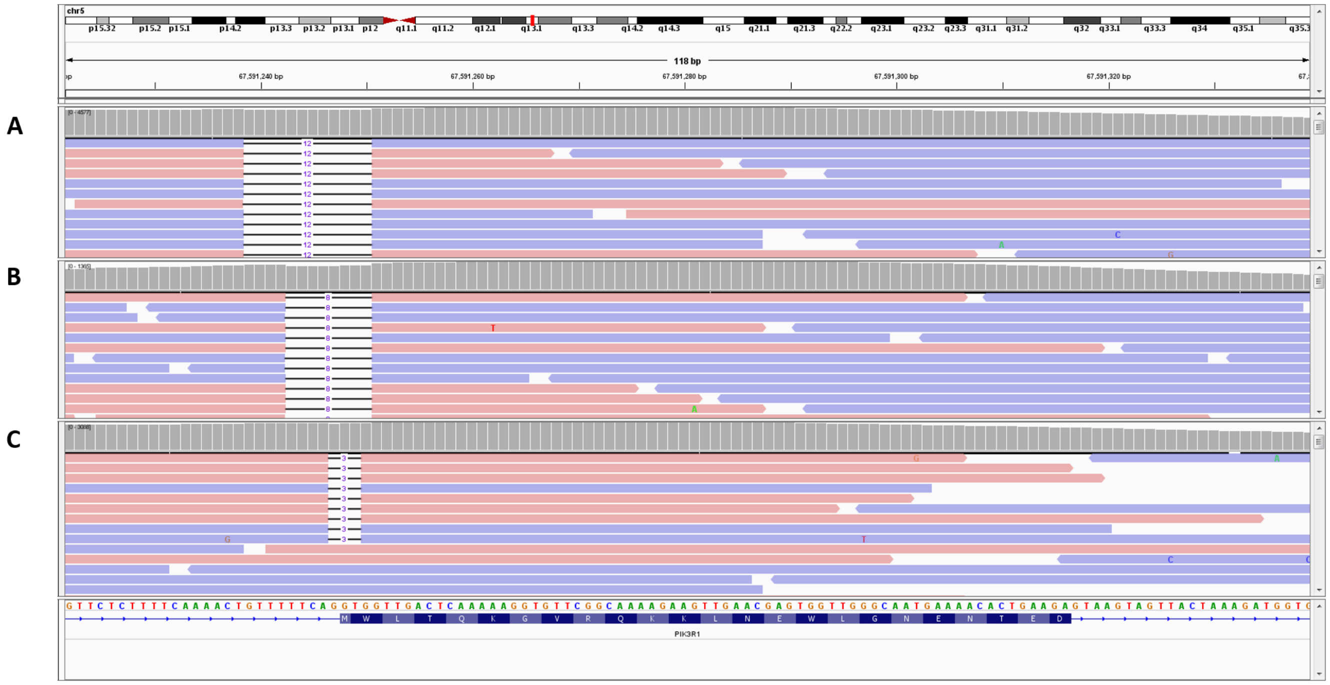

PIK3R1 NM_181523.3 Exon 14

Supplementary Figure 2: Splice-site Variants among Individuals with Vascular Malformation/Overgrowth as Visualized in Aligned Sequence Reads

Visualization within the Integrative Genomics Viewer (IGV)[1] of aligned reads in the BAM file derived from high-depth next-generation sequencing following target capture of the affected tissue of three individuals. A. Individual 6 harboring a 12 bp splice-site variant, B. Individual 7 harboring an 8 bp splice-site variant, C. Individual 8 harboring a 3 bp splice-site variant. These splice acceptor variants are predicted to encode an in-frame exon 14 skipping event, a described consequence of variants of this type in cancer.
